# Expression of the Folate Receptor Proteins FOLR1 and FOLR2 in Correlation With Clinicopathological Variables of Gastric Cancer

**DOI:** 10.7759/cureus.61032

**Published:** 2024-05-24

**Authors:** S Anitha, Raveendran Ramasamy, Rajesh Nachiappa Ganesh, Biswajit Dubashi

**Affiliations:** 1 Department of Pharmacology, Jawaharlal Institute of Postgraduate Medical Education and Research, Puducherry, IND; 2 Department of Pathology, Jawaharlal Institute of Postgraduate Medical Education and Research, Puducherry, IND; 3 Department of Medical Oncology, Jawaharlal Institute of Postgraduate Medical Education and Research, Puducherry, IND

**Keywords:** gastric cancer, qrt-pcr, ihc, folr2, folr1

## Abstract

Introduction: Gastric cancer (GC) remains a leading cause of cancer-related mortality worldwide, owing to its aggressive nature and poor prognosis. The role of folate receptors, particularly folate receptor 1 (FOLR1) and folate receptor 2 (FOLR2), in cancer has been increasingly recognized due to their overexpression in various malignancies including gastric cancer, and its potential implications in cancer progression, treatment resistance and as therapeutic targets.

Objective: To evaluate the expression patterns of FOLR1 and FOLR2 in GC patients' tissue and blood specimens and to correlate these patterns with clinicopathological variables.

Methods: A total of 58 gastric cancer patients were enrolled at the Regional Cancer Centre (RCC) from March 2017 to March 2020. Immunohistochemical analysis was performed to examine the expression of FOLR1 and FOLR2 in formalin-fixed paraffin-embedded (FFPE) tissue samples. Quantitative reverse transcription polymerase chain reaction (qRT-PCR) was performed to analyze FOLR1 and FOLR2 expression in blood samples. Statistical analyses were conducted using chi-square tests, independent T-tests, and Kaplan-Meier survival analysis.

Results: FOLR1 and FOLR2 were overexpressed in 82.76% and 70.69% of gastric cancer tissues, respectively. High expression levels of FOLR1 were significantly associated with the diffuse type of gastric cancer (p<0.005). qRT-PCR showed significant overexpression of FOLR1 in gastric cancer blood samples compared to control samples, with a median fold change of approximately 14.18 times. Conversely, FOLR2 was significantly underexpressed in gastric cancer samples, with a fold change of 0.30. However, no significant correlation was found between FOLR2 expression and the clinicopathological features. The overall survival analysis did not show a significant difference in survival rates based on the expression levels of FOLR1 and FOLR2.

Conclusions: This study highlights the differential expression patterns of FOLR1 and FOLR2 in gastric cancer and underscores the complexity of their roles in cancer biology. While FOLR1 shows potential as a biomarker for gastric cancer due to its overexpression, further studies are needed to fully elucidate the therapeutic and prognostic implications of folate receptors in gastric cancer.

## Introduction

Gastric cancer (GC) is a complex and multifactorial disease, with genetic and environmental factors influencing its pathogenesis [1]. GC is a high-aggressive cancer with a heterogeneous nature that continues to be a global health issue. The incidence of gastric cancer varies greatly in the different geographic regions and is more common in males than females; Eastern Asia, Eastern Europe, and South America have the highest rates, while North America and parts of Africa have the lowest [2]. It is the second most common cause of cancer-related deaths in India with incidence particularly high in the northeastern states, such as Mizoram, and is comparable to high-incidence areas globally [3,4]. Despite numerous breakthroughs in gastric cancer therapeutics, many patients suffer recurrence and eventually advance to metastatic stage.

Folic acid, also known as folate, is a B vitamin necessary for several biological processes such as cell division, DNA synthesis, and DNA repair [5]. Folates are required for single carbon transfer processes in eukaryotic cells, including the conversion of homocysteine to methionine and a variety of steps in de novo nucleotide synthesis [6]. Folate receptors (FRs) are cysteine-rich glycosylphosphatidylinositol (GPI)-anchored glycoprotein present on the cell surface, comprising three isoforms in humans that encode functional folate receptors namely FOLR1, FOLR2, and FOLR3 (also known as hFRα, hFRβ, and hFRγ, respectively) [7]. FRs are expressed in relatively low concentrations in normal cells but are mostly overexpressed on the cell surface of cancer cells, to compensate for folate requirements of rapidly proliferating cells. However, with the exception of a rare subtype of activated macrophage, normal cells in the circulatory system very rarely express FR [8]. Folate receptor alpha (FRα) was the most abundant and overexpressed isoform in cancer tissue compared to normal tissue [9].

The inconsistent and limited findings regarding the expression patterns of folate receptors in gastric cancer necessitated the initiation of the present study. No study has investigated the expression of both FOLR1 and FOLR2 in the same group of patients with GC. Our study objective was to evaluate the expression pattern of FOLR1 and FOLR2 in gastric cancer patients' tissue and blood specimens and correlate the results with the clinicopathological parameters. There is a lack of comprehensive studies investigating the roles of FOLR1 and FOLR2 in gastric cancer. This study addresses this gap, potentially contributing valuable data to the field, and influencing future research directions.

## Materials and methods

Patient characteristics

A total of 58 gastric cancer patients were enrolled at the Jawaharlal Institute of Postgraduate Medical Education and Research (JIPMER) Regional Cancer Centre (RCC) from March 2017- March 2020. The patient’s demographic characteristics are given in Table 1.

**Table 1:** Baseline Demographics and Clinico-Pathological Features of Gastric Cancer Patients (N=58) NACT- neoadjuvant chemotherapy, EOX - epirubicin, oxaliplatin, and capecitabine, GEJ - gastroesophageal junction

Clinical characteristics	Frequency (%) N=58	
Age (Median with range)	52 (21-76)	
Gender		
Female	23 (39.7)	
Male	35(60.3)	
Duration of Symptoms (Months)	3 (1-18)	
Comorbidity		
Yes	8(13.8)	
No	50(86.2)	
Stage		
Locally advanced	26(44.8)	
Metastasis	32(55.2)	
Site of Metastasis		
Liver	16(50.0)	
Nodal	13(40.6)	
Non-Peritoneal	1(3.1)	
Ovary	2(6.3)	
Site of tumour		
GEJ/Cardia/Body	23(39.7)	
Antrum/Pylorus	35(60.3)	
Diffuse vs. Intestinal		
Diffuse	22(37.9)	
Intestinal	18(31.0)	
Not available	18(31.0)	
Signet vs. Non- Signet		
Signet	9(40.9)	
Non-Signet	13(59.1)	
Chemotherapy	EOX	EOX+Aspirin
NACT	12(100)	-
Adjuvant	15(100)	-
Palliative	23 (92.0)	2 (8.0)

Ethics statement

All gastric cancer tissue and blood samples were collected in accordance with the protocol approved by the Institutional Ethics Committee (JIP/IEC/2017/284). Written informed consent was obtained from all participants prior to sample collection. The samples were used in strict compliance with the approved protocol, ensuring adherence to ethical standards and regulatory guidelines.

Immunohistochemical analysis

An immunohistochemistry (IHC) assay was performed to examine the expression of FOLR1 and FOLR2 in the formalin-fixed paraffin-embedded (FFPE) tissue sample of gastric cancer patients. The expression was detected using an anti-folate binding protein antibody from Abbexa Ltd. (cat No. abx421111, abx139453; Cambridge, UK). All specimens were fixed with 10% formalin, embedded in paraffin, and rinsed well in distilled water after formalin fixation. The tumor sections were cut into 3 µm sections and fixed on glass slides. FFPE samples were deparaffinized with three changes of xylene and rehydrated in a series of alcohol followed by 5% H_2_0_2 _to inactivate endogenous peroxidases. Antigen retrieval was achieved by placing the slides in a pressure cooker containing citrate buffer for heat-induced epitope retrieval until temperature reaches 95^o^-100^o^ C, then cooled in buffer for 20 minutes to 90^o^ C followed by five minutes of rinse in running water.

Slides were rinsed well and incubated for one hour at 37^o ^C with primary antibody. After washing twice with buffer, the sections were incubated with secondary antibody for 45 minutes.

To visualize peroxidase activity, sections were incubated in 1 mg/mL of 3,3,-diaminobenzidine (DAB) for 15 minutes. Subsequently, sections were counterstained with hematoxylin, washed and mounted with permanent mounting media, and coverslipped. Lung carcinoma and spleen tissue sections were taken as positive control for FOLR1 and FOLR2 respectively as suggested by the manufacturer. Chronic superficial gastritis was taken as a negative control for both markers ensuring specificity and sensitivity of the staining process.

Validation of folate alpha and beta expression detection

Multiple dilutions of the primary antibody (1:50, 1:75, 1:100, 1:200, 1:250, 1:400) were tested to determine the optimal concentration that provides the distinct staining with minimal background interference. Different incubation times (20 min, 30 min, 60 min) were evaluated to identify the ideal time frame that enhances antibody-antigen interactions while preventing non-specific binding. The antigen retrieval process was refined by experimenting with citrate buffer solutions at different pH levels (pH = 6.0 and pH = 9.0). The optimized protocol ensured consistent staining quality across all samples, facilitating reliable comparative analysis of FOLR1 and FOLR2 expression in gastric cancer tissues.

IHC scoring

Digital images of the stained sections were captured and evaluated for membrane staining intensity, categorized as negative (0), weak (+), moderate (++), and strong (+++). They were analyzed under 4×, 10×, 20×, and 40× objectives. Thick membranous staining, already visible at 4× and confirmed at 10× objective was scored as 3+ staining. 2+ staining: thinner and weaker in intensity than 3+ staining, visible at 10× and confirmed at 20× objective, while 1+ staining done at 20× and/or 40× objective for interpretation, and reflected very thin, weak staining of membrane.

RNA extraction and reverse transcription

Total RNA extraction from blood was performed using Direct-Zol RNA Miniprep (Zymo Research, Orange, CA, USA) as instructed by the manufacturer. Just briefly, ethanol was added to the lysate after the sample lysis for optimal binding. The lysate was then thoroughly washed and loaded onto the Zymo-Spin™ silica membrane for RNA binding, followed by DNAase treatment. The RNA was water-eluted after repeated washing with buffers. The concentration and purity of eluted RNA solutions were then determined using Nano-Biospectrophotometer (Eppendorf, Hamburg, Germany). 1 µg of total RNA was used for reverse transcription following the instructions of PrimeScript 1st strand cDNA Synthesis Kit (#6110A; Takara Bio Inc., Shiga, Japan).

Quantitative RT-PCR

Thermal cycling was done in a reaction volume of 20 μL in triplicate using SYBR® Premix Ex Taq™ (Takara Bio), followed by melting curve analyses. All primer sets were purchased from Sigma Aldrich (St. Louis, MO, USA). The primer sets are provided in Table 2. Amplification data for all samples were collected from QuantStudio™ 3 Real-Time PCR System (Applied Biosystems, Waltham, MA, USA). Data were normalized to the endogenous control glyceraldehyde 3-phosphate dehydrogenase (GAPDH) and messenger RNA (mRNA) abundance was calculated using the 2^-ΔΔCT^ method.

**Table 2:** List of the primers used for quantitative polymerase chain reaction (qPCR) FOLR1 - folate receptor 1, FOLR2 - folate receptor 2, GAPDH - glyceraldehyde 3-phosphate dehydrogenase

Primers	Forward (5′–3′)	Reverse (5′–3′)
FOLR1	AAAGAGCGGGTACTGAACGTG	CGCACTTGTTAAACCCTGAAGTC
FOLR2	CGCGTTGTCTACCCTGTACC	GAGCTGAACCTCCGTTGCT
GAPDH	TCCAAAATCAAGTGGGGCGA	TGATGACCCTTTTGGCTCCC

Statistical analysis

Categorical variables were expressed as frequencies and percentages. Continuous variables like age and duration of symptoms were expressed as median and range values. The expressions of FOLR1 and FOLR2 proteins, determined by IHC, were compared with selected clinicopathological parameters using the chi-square test, to assess whether the observed frequencies of FOLR1 and FOLR2 expressions differ significantly from the expected frequencies across the parameters. Independent T-test is used to compare the fold change between FOLR1 and FOLR2 in the blood of gastric cancer patients. The overall survival analysis was performed using the Kaplan-Meier estimate, a non-parametric statistical method for estimating the survival function from lifetime data. A p-value less than 0.05 (p < 0.05) was considered statistically significant for all the analyses performed. For the statistical analysis, Statistical Package for Social Sciences (SPSS) version 21 (IBM Corp., Armonk, NY, USA) was used.

## Results

**Figure 1:**
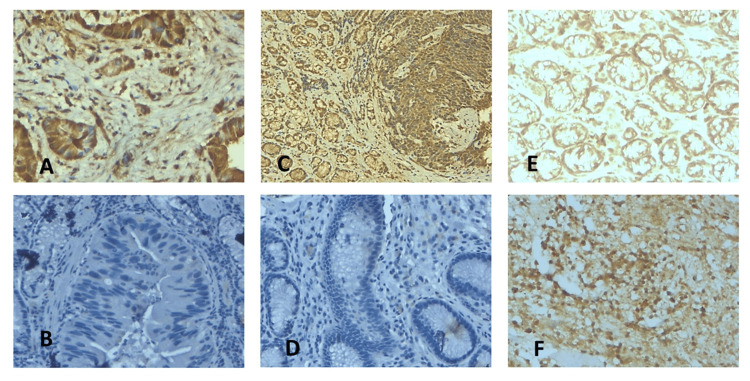
Immunohistochemistry Analysis of Folate Receptor 1 (FOLR1) and Folate Receptor 2 (FOLR2) Expression in Gastric Cancer Tissue Samples A, B) Positive FOLR1 and FOLR2 expression in gastric cancer tissues, characterized by intense brown staining. B, D) Negative FOLR1 and FOLR2 expression in gastric cancer tissues. E, F) Positive control for FOLR1 and FOLR2

The expression of folate receptor 1 and folate receptor 2 was examined immunohistochemically in 58 gastric cancer samples and 10 normal gastric tissues. In all cases of the control group in normal gastric mucosa, expression of these proteins was absent. Microscopic analysis demonstrated that positive expression of FOLR1 and FOLR2 proteins in tumor cells was present in 48 (82.76%) and 41 (70.69%) out of 58 patients, respectively. In tumor cells of all cases, the expression of these proteins was observed both in the cell membrane and cytoplasm (Figure 1).

A comprehensive overview of various characteristics observed in a study population is provided in Table 1. The median age was 52 years, with ages ranging from 21 to 76 years, showing a broad age distribution. The majority of patients were male (35; 60.3%), and the median symptom duration before diagnosis was three months. A small percentage of patients (eight; 13.8%) had comorbidities, while the majority (50; 86.2%) did not. Nearly half of the patients (26; 44.8%) were at a locally advanced stage of cancer, and 32 (55.2%) had metastases, with the liver being the most common metastasis site (16; 50.0%). The primary tumor locations were mainly in the antrum or pylorus (35; 60.3%). Histologically, the distribution between diffuse (22; 37.9%) and intestinal (18; 31.0%) types was nearly equal, with signet ring cell carcinoma present in nine (40.9%) of the cases. EOX (epirubicin, oxaliplatin, and capecitabine) was the most commonly used chemotherapy regimen across all groups with 12 (100%), 15 (100 %) of patients in the neoadjuvant chemotherapy (NACT) and adjuvant groups receiving this combination, and 23 (92%) in the palliative group. 

In a cohort of 58 gastric cancer patients, immunohistochemical analysis was used to assess the expression of FOLR1 and FOLR2 and correlate these findings with various clinical and pathological variables such as age, gender, cancer stage, tumor site, liver involvement, and histological type (Table 3). The study revealed high expression rates of FOLR1 and FOLR2 in gastric cancer tissues, at 48 (82.76%) and 41 (70.69%) respectively. The statistical analysis demonstrated significant differences in the expression of FOLR1 and FOLR2 between the diffuse and intestinal histological types of gastric cancer. This suggests that the histological type may influence the expression of these folate receptors. However, no statistically significant associations were found between these expressions and the other variables analyzed. This lack of significant findings in the other variables may be attributed to the relatively small sample size of the study, which can limit the statistical power to detect true associations.

**Table 3:** Folate Receptor 1 (FOLR1) and Folate Receptor 2 (FOLR2) expressions compared with the clinical and pathological variables of the participants p < 0.001^**^ GEJ - gastroesophageal junction, PO - positive, NEG - negative

Variable	N=58	Category	FOLR1(PO-1/NEG-0)			FOLR2(PO-1/NEG-0)		
			0	1	p-value	0	1	p-value
Age	58	Median with range	52(36-65)	52(21-76)	0.62	55(36-76)	52(21-70)	0.34
Gender	23	Female	4(40)	19(39.6)	1.00	8(47.1)	15(36.6)	0.60
	35	Male	6(60)	29(60.4)		9(52.9)	26(63.4)	
Stage	26	Locally advanced	6(60.0)	20(41.7)	0.32	9(52.9)	17(41.5)	0.56
	32	Metastasis	4(40.0)	28(58.3)		8(47.1)	24(58.5)	
Site of tumour	23	GEJ/Cardia/ Body	5(50.0)	18(37.5)	0.50	7(41.2)	16(39.0)	1.00
	35	Antrum/Pylorus	5(50.0)	30(62.5)		10(58.8)	25(61.0)	
Diffuse vs. Intestinal	22	Diffuse	3(30.0)	19(90.5)	0.001**	7(58.3)	15(53.6)	1.00
	18	Intestinal	7(70.0)	2(9.5)		5(41.7)	13(46.4)	
Signet vs. Non-Signet	9	Signet	2(50.0)	7(38.9)	1.00	4(57.1)	5(33.3)	0.38
	13	Non-Signet	2(50.0)	11(61.1)		3(42.9)	10(66.7)	

The fold change and average delta cycle threshold (ΔCT) values for each RNA marker in the blood of gastric cancer samples, along with the normal samples are provided in Table 4. The ΔCT is inversely proportional to the amount of target RNA in the sample; thus, a lower ΔCT indicates higher expression. Folate alpha shows a significant overexpression in gastric cancer blood compared to control samples, with a median fold change of approximately 14.18 times. This substantial increase, coupled with a very low p-value (0.002), suggests a potentially critical role of FOLR1 in the pathophysiology of gastric cancer. Folate beta, in contrast, is significantly underexpressed in gastric cancer samples, with a fold change of 0.30. The p-value of 0.022 indicates that this difference is statistically significant, although the role of decreased folate 2 expression in cancer development or progression would require further investigation.

**Table 4:** Folate expression in blood samples of gastric cancer as compared with control samples

Gene	Gastric cancer ΔCT N=27	Normal ΔCT N=4	p-value	Fold change (2^−ΔΔCT^) N=31
Folate 1	11.67±2.04	15.20±0.14	0.002^**^	14.18(0.27-84.57)
Folate 2	5.15±2.17	2.48±0.12	0.022^*^	0.30(0.01-1.44)

**Figure 2:**
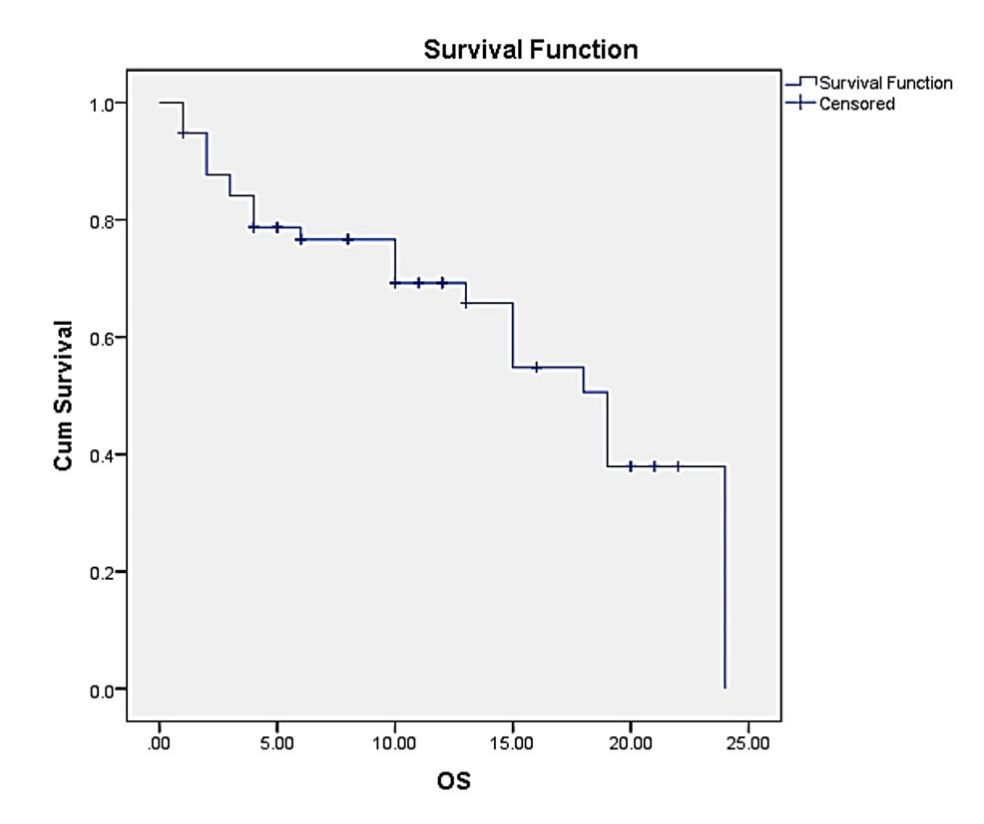
Survival – Overall survival (OS) of study participants Median duration of follow-up = 10 (1-24) months Median OS = 19 (15.18-22.83) months

Kaplan-Meier survival curve was constructed for the overall survival (OS) of gastric cancer patients. The OS of 58 gastric cancer patients over a follow-up period ranging from one to 24 months is shown in Figure 2. The median OS time was 19 months, with a 95% confidence interval of 15.18 to 22.83 months. The analysis included a total of 28 events (deaths) and 30 censored observations, accounting for 51.7% of the cohort.

**Figure 3:**
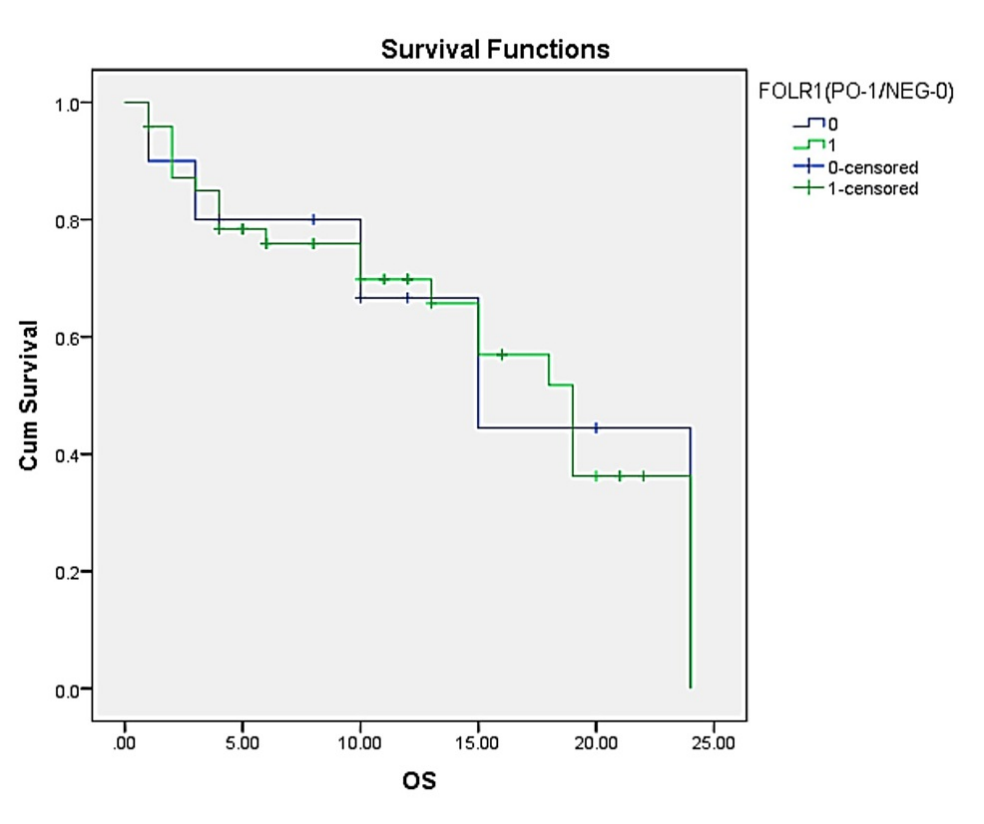
Kaplan-Meier Survival Curve Stratified by Folate Receptor 1 (FOLR1) Expression OS - overall survival, PO - positive, NEG - negative

Overall survival of the study participants was stratified based on the expression of FOLR1 and FOLR2: those with positive (PO) expression and those with negative (NEG) expression. Figure 3 includes data for a total of 58 patients, with 48 showing positive FOLR1 expression and 10 showing negative expression. The number of events, which typically refers to deaths in the context of survival analysis, is 19 for the FOLR1-PO group and 15 for the FOLR1-NEG group. The percentage of censored data is five (50.0%) for the FOLR2-PO group and 25 (52.1%) for the FOLR1-NEG group. Censoring occurs when the study ends before an event is observed, or if a participant is lost to follow-up, indicating that over half of the patients in each group did not have the event occur during the study period. The median OS in FOLR1-PO is 19 months, with a confidence interval of 15.21 to 22.79 months whereas in FOLR1-NEG it is 15 months, with a confidence interval of 5.70 to 24.30 months. Although there are differences in survival times and the proportion of censored data between the two groups, there was no statistical significance in survival between the groups.

**Figure 4:**
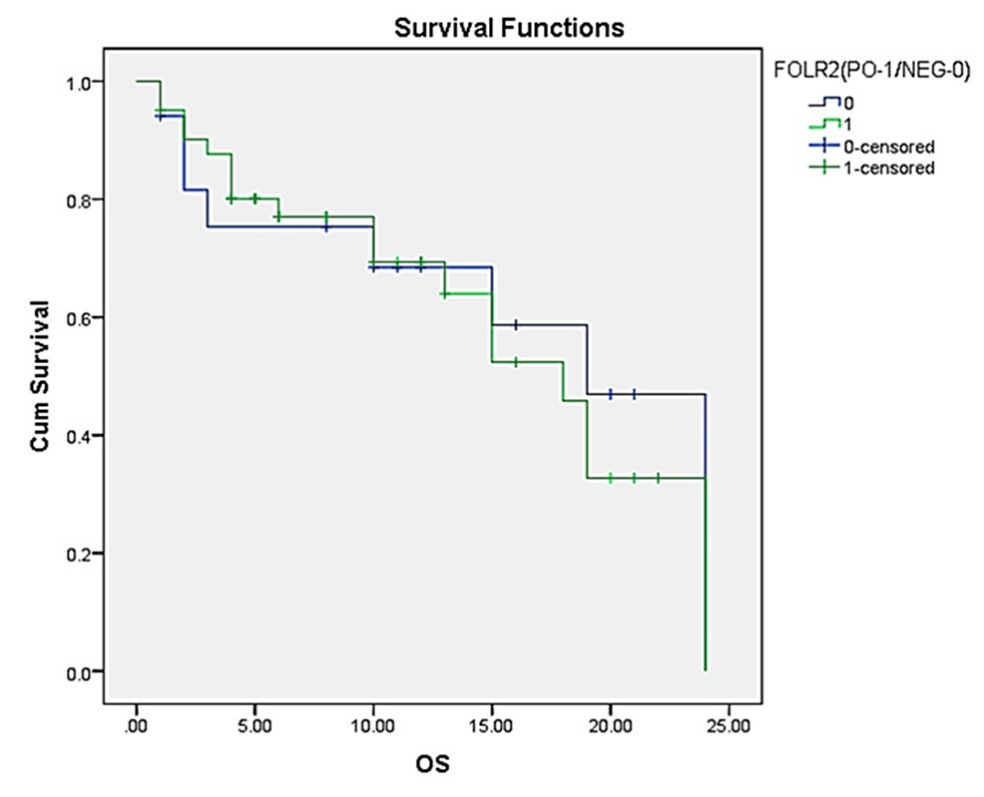
Kaplan-Meier Survival Curve Stratified by Folate Receptor 2 (FOLR2) Expression OS - overall survival, PO - positive, NEG - negative

Among 58 patients, 41 patients had positive FOLR2 expression and 17 showed negative protein expression. The median OS is 18 (13.86-22.14) months for the FOLR2-PO group and 19 (14.47-23.53) months for FOLR2-NEG group The p-value associated with the comparison between the two groups is 0.70, suggesting that there is no statistically significant difference in survival between patients with positive and negative FOLR2 expression (Figure 4).

## Discussion

Gastric cancer remains a formidable challenge in oncology, owing to its aggressive nature and significant mortality rates globally [10,11]. The quest for early detection markers and effective therapeutic targets is paramount to improving patient outcomes. In this context, our study focused on the immunohistochemical expression of FOLR1 and FOLR2 in gastric cancer patients, aiming to elucidate their roles in the disease's pathogenesis and potential as prognostic markers or therapeutic targets [12].

The expression of folate receptors, particularly FOLR1 and FOLR2, has been increasingly recognized as a significant factor in the development and progression of GC [13,14]. These receptors, which are overexpressed in various malignancies, including GC, mediate the cellular uptake of folate, a B vitamin essential for DNA synthesis, repair, and methylation [15,16]. 

Immunohistochemical analysis demonstrated presence of folate receptors in the cancer cells across the examined samples, as evidenced by the substantial number of positive results. This observation aligns with the known overexpression of folate receptors in various malignancies, which is thought to support the increased metabolic demands of rapidly proliferating cancer cells [17]. The significant differences observed between the histological subtypes of gastric cancer, specifically diffuse and intestinal types, in relation to FOLR1 and FOLR2 expressions, align with the growing body of evidence that suggests molecular heterogeneity within gastric cancer subtypes [18]. The high percentage of positivity for FOLR1 and FOLR2 expressions in gastric cancer tissues (82.76% and 70.69%, respectively) underscores the potential role of these receptors in gastric cancer biology. However, despite the clear indication of folate receptor positivity, our statistical analysis did not show a significant correlation between folate receptor expression and clinicopathological variables such as tumor stage, tumor site, or lymph node involvement. This lack of statistical significance might be due to the relatively small sample size of our study, which could limit the power to detect such associations.

In the blood samples of gastric cancer patients, FOLR1 was overexpressed, with a median fold change of 14.18 times higher than in normal samples, a difference that is statistically significant. This finding aligns with previous research indicating that FOLR1 is overexpressed in various malignancies and is associated with accelerated cancer progression and poor patient prognosis [14]. Conversely, FOLR2 expression was markedly underexpressed, with a fold change of 0.30 compared to control blood samples. FOLR2, like FOLR1, is involved in folate uptake but has a different tissue distribution and is expressed in tissue-resident macrophages. The underexpression of FOLR2 could reflect alterations in the tumor microenvironment or immune response dynamics in gastric cancer [19].

According to recent research, FOLR1 is involved in a high number of intracellular signaling networks, many of which impact cancer cells, namely JAK-STAT3, ERK1/2, and as a transcription factor signaling pathways [13]. A study by Gonzalez et al. explored the diagnostic potential of FOLR1 in gastric cancer using a liquid biopsy technique, emphasizing its overexpression and diagnostic relevance [20]. Our findings indicate a significant overexpression of FOLR1 and FOLR2 in gastric cancer tissues compared to normal gastric mucosa, aligning with previous studies that highlighted the aberrant expression of folate receptors in malignancies [16,21].

Overall, our findings support the idea that FOLR1 and FOLR2 could serve as potential targets for the development of novel therapies for gastric cancer. Inhibition of these receptors could lead to the selective targeting and killing of cancer cells while sparing normal cells. The overexpression of FOLR1 in gastric cancer opens avenues for targeted therapeutic strategies [22,23]. Folate receptor-targeted therapies, such as folate-drug conjugates and folate receptor-specific antibodies, have shown promise in preclinical models. Further studies are needed to investigate the feasibility and effectiveness of FR-targeted therapies in gastric cancer treatment.

Limitations and future directions

While our study provides important insights into the expression and implications of FOLR1 and FOLR2 in gastric cancer, it has certain limitations. The small sample size may impact the generalizability of our findings. To confirm our results and further explore the mechanistic roles of FOLR1 and FOLR2 in the progression of gastric cancer, future research involving larger, prospective cohorts is necessary. Furthermore, the exploration of FOLR1 and FOLR2 expression in response to treatment and their impact on patient survival could offer deeper insights into their potential as therapeutic targets and prognostic markers.

## Conclusions

In conclusion, our study highlights the significant overexpression of FOLR1 and FOLR2 in gastric cancer and its association with adverse clinicopathological features, particularly for FOLR1. These findings underscore the potential of FOLR1 and FOLR2 as biomarkers for gastric cancer prognosis and as targets for folate receptor-directed therapies. Further research is needed to fully elucidate their roles in gastric cancer pathogenesis and therapeutic response, paving the way for improved diagnostic and treatment strategies.

## References

[1] Rawla P, Barsouk A (2019). Epidemiology of gastric cancer: global trends, risk factors and prevention. Prz Gastroenterol.

[2] Machlowska J, Baj J, Sitarz M, Maciejewski R, Sitarz R (2020). Gastric cancer: epidemiology, risk factors, classification, genomic characteristics and treatment strategies. Int J Mol Sci.

[3] Dikshit RP, Mathur G, Mhatre S, Yeole BB (2011). Epidemiological review of gastric cancer in India. Indian J Med Paediatr Oncol.

[4] Sathishkumar K, Chaturvedi M, Das P, Stephen S, Mathur P (2022). Cancer incidence estimates for 2022 & projection for 2025: Result from National Cancer Registry Programme, India. Indian J Med Res.

[5] Chen C, Ke J, Zhou XE (2013). Structural basis for molecular recognition of folic acid by folate receptors. Nature.

[6] Wibowo AS, Singh M, Reeder KM (2013). Structures of human folate receptors reveal biological trafficking states and diversity in folate and antifolate recognition. Proc Natl Acad Sci U S A.

[7] Fang PW, Lin YC, Fan SY, Panja A, Xu SQ, Lee SH, Tan KT (2023). Protein-labeling fluorescent probe for folate receptor α. Anal Chem.

[8] Xue Y, Cong W, Xie S, Shu J, Feng G, Gao H (2018). Folate-receptor-positive circulating tumor cells as an efficacious biomarker for the diagnosis of small pulmonary nodules. J Cancer Res Ther.

[9] Kim M, Pyo S, Kang CH, Lee CO, Lee HK, Choi SU, Park CH (2018). Folate receptor 1 (FOLR1) targeted chimeric antigen receptor (CAR) T cells for the treatment of gastric cancer. PLoS One.

[10] Bray F, Ferlay J, Soerjomataram I, Siegel RL, Torre LA, Jemal A (2018). Global cancer statistics 2018: GLOBOCAN estimates of incidence and mortality worldwide for 36 cancers in 185 countries. CA Cancer J Clin.

[11] Rona KA, Schwameis K, Zehetner J (2017). Gastric cancer in the young: an advanced disease with poor prognostic features. J Surg Oncol.

[12] Song Z, Wu Y, Yang J, Yang D, Fang X (2017). Progress in the treatment of advanced gastric cancer. Tumour Biol.

[13] Nawaz FZ, Kipreos ET (2022). Emerging roles for folate receptor FOLR1 in signaling and cancer. Trends Endocrinol Metab.

[14] Zeng CD, Jin CC, Gao C, Xiao AT, Tong YX, Zhang S (2022). Preoperative folate receptor-positive circulating tumor cells are associated with occult peritoneal metastasis and early recurrence in gastric cancer patients: a prospective cohort study. Front Oncol.

[15] Lee TY, Chiang EP, Shih YT, Lane HY, Lin JT, Wu CY (2014). Lower serum folate is associated with development and invasiveness of gastric cancer. World J Gastroenterol.

[16] Low PS, Henne WA, Doorneweerd DD (2008). Discovery and development of folic-acid-based receptor targeting for imaging and therapy of cancer and inflammatory diseases. Acc Chem Res.

[17] Zhang G, Zhang Q, Miao X (2005). Polymorphisms and mutations of the folate receptor-alpha gene and risk of gastric cancer in a Chinese population. Int J Mol Med.

[18] Ho SW, Tan P (2019). Dissection of gastric cancer heterogeneity for precision oncology. Cancer Sci.

[19] Samaniego R, Domínguez-Soto Á, Ratnam M, Matsuyama T, Sánchez-Mateos P, Corbí ÁL, Puig-Kröger A (2020). Folate receptor β (frβ) expression in tissue-resident and tumor-associated macrophages associates with and depends on the expression of PU.1. Cells.

[20] Gonzalez T, Muminovic M, Nano O, Vulfovich M (2024). Folate receptor alpha-a novel approach to cancer therapy. Int J Mol Sci.

[21] Yi YS (2016). Folate receptor-targeted diagnostics and therapeutics for inflammatory diseases. Immune Netw.

[22] Sakai H, Kawakami H, Teramura T (2021). Folate receptor α increases chemotherapy resistance through stabilizing MDM2 in cooperation with PHB2 that is overcome by MORAb-202 in gastric cancer. Clin Transl Med.

[23] Cao B, Liu L, Zhang R, Dong H, Shen J (2024). Sensitivity and specificity of folate receptor α-positive circulating tumour cells in gastric cancer. Postgrad Med J.

